# Eighth District Society of New York

**Published:** 1871-06

**Authors:** 


### EIGHTH DISTRICT SOCIETY OF NEW YORK.

We attended the meeting of this society in Buffalo, on the 9th and 10th of May, and found the members actively engaged in the cause of dental progress. About forty members were present, and all seemed to be earnest and cordial. Visitors received such a hearty welcome, that they almost forgot they were not members. A number of valuable essays were read, and the discussions were mostly of a high order.

Among the visitors was Dr. Bonwill, of Philadelphia, with his electric plugger. This instrument was practically tested in the presence of the society. Like many other inventions, it looks very simple now. Had a telegraph operator turned dentist, he would have thought of it at once. We have simply the to and fro motion of the ordinary galvano-electric machine, the hammer striking the plugger, and driving it the one-fiftieth of an inch, just before it is arrested by contact with the magnet. The blow is sufficiently strong, and its force may be increased or diminished by varying the length of the stroke, which is done by a set-screw. The strokes are very rapid, and they may be at once suspended, by moving a slide with the index finger. The plugger may be rotated with the thumb, without changing the general position of the instrument.

A social meeting at Dr. Southwick's in the evening, was very enjoyable, and doubtless, added much to the pleasures of the occasion. If Mrs. S. and daughter intended to make us forget the cares incident to professional life, they may feel flattered with their success.

But, after all, this society should either add a day to each meeting, or forego its usual social enjoyment. A social meeting can be gotten up at any time; but when forty dentists get together, each one should try to learn all that the other thirty-nine can teach him.

But we can not do justice to the meeting. Those who should have been there, but were not, can have no idea of the loss they have sustained. Their patients will one day find out that they are behind the times, and such discovery will be expensive.

